# Comparison of Commercial Calcium Hydroxide Pastes for Prolonged Antibacterial Effect using a Colourimetric Assessment

**DOI:** 10.3390/ma11030348

**Published:** 2018-02-27

**Authors:** Yu-Yao Teoh, Basil Athanassiadis, Laurence J. Walsh

**Affiliations:** 1 School of Dentistry, The University of Queensland, Herston, QLD 4006, Australia; l.walsh@uq.edu.au; 2 Private Dental Practice, Annerley, QLD 4103, Australia; basildent@bigpond.com

**Keywords:** calcium hydroxide, hydroxyl ions, polyethylene glycol, solvent, vehicle, intracanal medicament, pastes

## Abstract

The anti-microbial activity of calcium hydroxide pastes used in endodontics is dependent on establishing high levels of hydroxyl ions in dentine. This study investigated hydroxyl ion diffusion from different commercial calcium hydroxide pastes using a novel colourimetric method. In this method, human tooth roots were stained with anthocyanin dye, which changed their colour according to the local pH conditions. Prepared root canals were filled with pastes formulated with the vehicle of water (Pulpdent™, Calasept Plus™), polyethylene glycol (PEG) (Calmix™) or a mixture of water, PEG and ibuprofen (Odontocide™). The changes in dye colour at fixed distances from the canal wall were monitored using standardised digital photography over a period of 3 weeks. A repeated measures analysis tracked changes in each root from baseline. Release of hydroxyl ions varied between the different commercial compositions containing water or PEG as solvents. The colour changes in the dentine, due to released hydroxyl ions, were greatest and more prolonged for completely non-aqueous compositions, when using PEG 400 as the vehicle. When water was present in the product, the duration of the pH changes was shorter. This was attributed to the presence of hydroxyl ions in the water (the common-ion effect) and a more vigorous buffering of hydroxyl ions by dentine proteins.

## 1. Introduction

Calcium hydroxide medicament pastes are used widely in endodontics because hydroxyl ion release exerts broad spectrum antimicrobial activity against most pathogens found in the root canal. Hydroxyl ions can penetrate biofilms, inhibit the inflammatory actions of endotoxins and help to dissolve organic tissues [1,2,3]. Traditionally, calcium hydroxide pastes have been prepared using water as the solvent, with Pulpdent™ paste (Pulpdent Corporation, Watertown, MA, USA) having been on the market since 1947.

The release of hydroxyl ions from calcium hydroxide pastes is the key factor responsible for antimicrobial activity. Due to the presence of free hydroxyl ions within the water itself, the common-ion effect limits the maximum solubility of calcium hydroxide to 0.159 g/100 mL (0.16%) at 25 °C, which also reduces with increasing temperature [4]. Most commercial calcium hydroxide pastes, using a water base, contain excess calcium hydroxide content above the solubility limit. This excess can act as a thickening agent but does not contribute greatly to the therapeutic effects [1].

Several agents other than water have been used alone or in combination with water, as alternative vehicles for calcium hydroxide pastes, in order to modify the viscosity of the paste. For example, Calen PMCC™ contains polyethylene glycol (PEG) 400, paramonochlorophenol and camphor. The choice of the PEG 400 in Calen PMCC™ was purely as a viscosity enhancer, while the latter ingredients were included to improve the antibacterial properties [5]. Recently, it has been found that placing calcium hydroxide into a water-free composition, using PEG 400 in conjunction with high molecular forms of PEG as a vehicle, enhances the dissolution of calcium hydroxide and improves hydroxyl ion release [6,7]. This concept is the basis for Calmix™, a product where enhanced anti-microbial properties are derived from an increased hydroxyl ion release, without the addition of paramonochlorophenol and camphor, as in earlier medicaments.

Other viscosity modifiers that have been used in calcium hydroxide pastes include glycerol and propylene glycol [2]. PEG 400 is also used in calcium hydroxide endodontic pastes, which contain ibuprofen (Odontocide™).

The aim of the present study was to assess the hydroxyl ion release of calcium hydroxide in various solvents, with an emphasis on hydroxyl ion penetration into dentine at different depths and the duration of its therapeutic effects.

## 2. Materials and Methods

### 2.1. Preparation of Dye Reference and Roots

The study followed a repeated measures design, with each root serving as its own experimental series starting from the baseline. This approach was used because biological variation between roots (size, colour and sclerosis) could be a confounding factor. To ensure reproducibility, the entire experiment was repeated twice, giving three sets of data for all medicament pastes.

**Figure 1:**
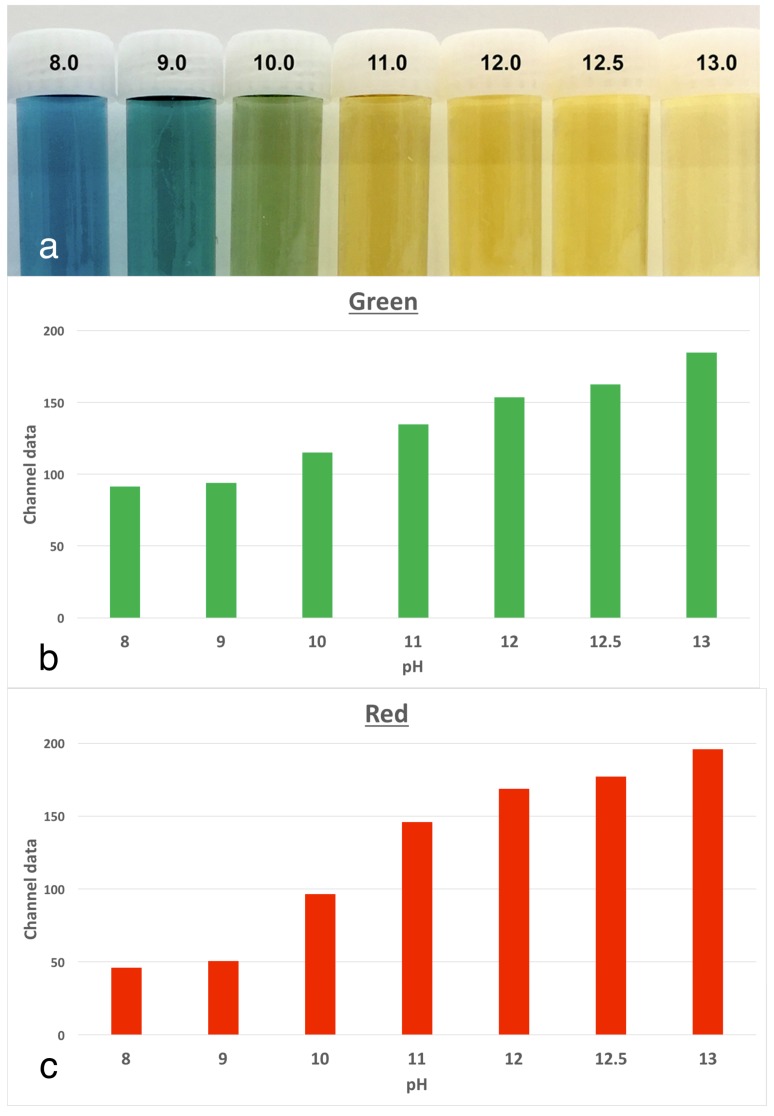
The colour changes in anthocyanin dye according to pH, using a series of buffers with the pH values from 8 to 13. Panel (**A**) shows the visual appearance of a solution of anthocyanin dye as the pH alters. Each vial has the same concentration of dye. Panels (**B**,**C**) show the digital pixel data for the pH standards of the red channel (**B**) and green channel (**C**). There is a consistent increase in both parameters as the pH rises.

The experimental model involved tracking changes in the colour of anthocyanin dye in stained roots, which were kept at physiological conditions. Anthocyanin is the pH indicator found in red cabbage and it undergoes multiple colour changes with the pH over the alkaline range. There are corresponding increases in both the red and green channels, as the colour changes. The use of anthocyanin staining was validated in a pilot study [6] where a series of buffers from pH 8.0 to 13.0 were prepared. The indicator dye was mixed in to serve as a reference for changes in pixel data in colour images (Figure 1).

Extracted human permanent teeth were collected from oral surgeons with the approval of the institutional ethics committee (Approval code #1311). A total of 23 single-canal teeth, free from intrinsic discolouration or translucency, were selected. Then, the crowns were removed at the cemento-enamel junction and the root face was smoothened to form a flat coronal surface using abrasive discs. After the patency of root canals was confirmed using a #8 K-file (Dentsply Maillefer, Ballaigues, Switzerland), they were then prepared with NiTi rotary instruments (ProTaper Next™, Dentsply Maillefer, Ballaigues, Switzerland) to a size X3 (apical preparation of #30 with variable taper). The root canals were irrigated alternately with 1% *w*/*v* sodium hypochlorite (Endosure Hypochlor 1% Solution™, Dentalife, Ringwood, Melbourne, Australia) and 15% *w*/*v* ethylenediaminetetraacetic acid (EDTA) with 0.85% *w*/*v* cetrimide (Endosure EDTA/C 15% Solution™, Dentalife, Ringwood, Melbourne, Australia) using syringes with side-vented needles. After a final irrigation, using Endosure EDTA/C for 2 min, the canals were dried with paper points and the roots placed in saline at room temperature for 24 h to ensure dissipation of any remnants of sodium hypochlorite.

### 2.2. Root Staining with Anthocyanin Dye

To stain the roots, a 3% solution of anthocyanin (Red Cabbage Jiffy Juice™ powder, Educational Innovations Inc., Bethel, CT, USA) was prepared in distilled water. The prepared roots were removed from the saline bath and dried with paper towels and paper points. Each canal was then irrigated with the prepared dye solution, before submerging the entire root into the dye for 48 h. This achieved a uniform purple stain throughout the root. The stained roots were dried with paper towels and mounted upright by using sticky wax on a holder, so that the coronal surface of the root could be photographed using a digital camera under fixed conditions (constant lighting, distance and exposure settings). A colour reference card and calibration ruler were placed adjacent to the root in the photograph to obtain a baseline image as a reference point from which subsequent changes could be measured.

### 2.3. Placement of Medicaments

The roots were assigned randomly to seven experimental groups of three roots each. Two roots served as an untreated control group in which no medicament was placed. The seven treatment groups were Calmix™ (Ozdent, Castle Hill, Sydney, Australia), Calasept Plus™ (Nordiska Dental AB, Ängelholm, Sweden), Pulpdent™ (Pulpdent Corporation, Watertown, MA, USA), Odontocide™ (Australian Dental Manufacturing, Kenmore, Brisbane, Australia), 10% calcium hydroxide in PEG 400, 20% calcium hydroxide in PEG 400 and PEG 400 as a vehicle control. Calcium hydroxide and PEG 400 were from Sigma-Aldrich (St Louis, MI, USA). The composition of the commercial products is given in Table 1.

The materials were injected into the root canal under positive pressure until excess was seen to extrude apically. After removing the excess material on the coronal and outer surface of each root, the apex was sealed with molten wax and each root was then placed into an individual Eppendorf™ tube, which was filled with anthocyanin dye solution to a level just below the coronal surface of the tooth. The coronal surface was left open to facilitate photographic records. The samples were then maintained at 37 °C in an incubator for 3 weeks.

### 2.4. Tracking Colour Changes in Root Dentine over Time

**Figure 2:**
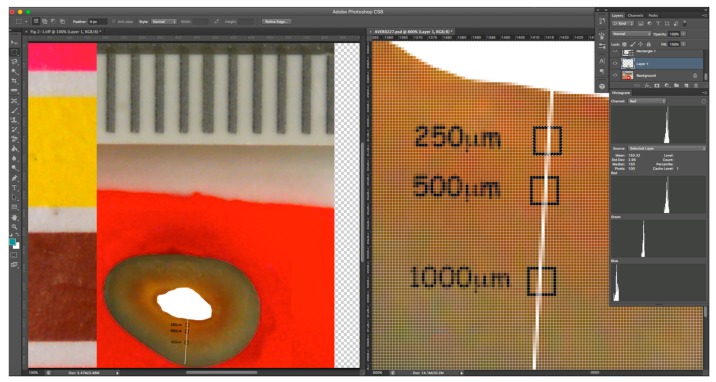
Screenshots showing the colour analysis method. In the example shown, the stained root had been treated with Calmix for 3 weeks. The right panel shows an 800% magnification of part of the left image. A white line was placed to ensure reproducible areas were elected between prospective images of the same root over time. Sample areas were taken at designated distances from the canal wall, as shown in the right panel. A total of 100 pixels in a 10 × 10 pixel selection area (annotated with a black square) were used for analysis, using the histogram tool.

Digital photographs of the coronal surface of the roots were taken after 1, 2, 3, 5, 7, 14, and 21 days, under standardised photographic conditions, with constant daylight-colour temperature lighting. The illumination jig ensured no reflection or glare on the target area being imaged. Image analysis was undertaken using Adobe Photoshop™ CS6 Extended software, to track changes in each root over time and to calculate the colour change in red and green channels from the baseline by using a repeated measures assessment. The colour channel analysis method used was similar to that employed in previous studies [8,9,10]. The selected areas of interest were located at 250 and 500 μm from the root canal walls in a straight line. The selected areas (10 × 10 pixels) were identified using the select tool (Figure 2) and were in the same location on sequential images of each root. To ensure the proper alignment, sequential images were overlaid on the baseline image using the layers function with reduced opacity, so that the same areas could be selected for analysis in each image. The pixel information was collected using the histogram tool of Adobe Photoshop™, which gave red and green values for the selected area for 8-bit colour, i.e., on a scale from 0 to 255. The extent of the change from the baseline was calculated for the three replicates from each group.

### 2.5. Analysis of Data

Data were analysed using Prism™ software version 7.0a (GraphPad Software Inc., La Jolla, CA, USA) by applying non-parametric analyses (ANOVA) for repeated samples as well as post-hoc comparisons between groups using Dunn’s test.

## 3. Results

**Figure 3:**
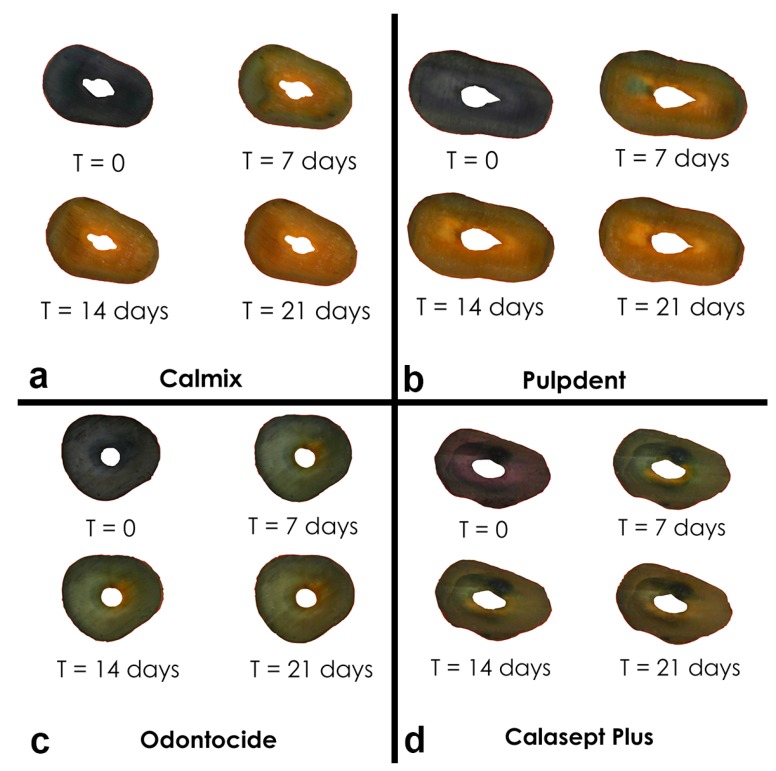
Image sequence of the coronal root surface of a set of roots treated with different commercial medicaments over time.

Composite images of roots were taken over time. An example is shown in Figure 3. Tracking the extent of colour change in the stained roots revealed obvious changes over the 3-week period of the experiment, with the characteristic change from a deep purple-blue baseline colour to a yellow colour for the dentine adjacent to the root canal. These changes were in line with the references prepared from anthocyanin in the buffered pH solutions [6]. From a visual perspective, the greatest colour changes in the anthocyanin stained roots were seen with Calmix™; however, there were no significant changes in the untreated roots with open canals or in the canals treated with 10% calcium hydroxide in PEG 400 or with PEG 400 only.

Quantitative analysis of changes in colour revealed positive changes in both the green and red pixel data at 250 μm from the canal wall. Each of the five calcium hydroxide materials indicated that elevations in the pH of the root dentine had occurred progressively over the first 7 days and then had begun to stabilise.

**Figure 4:**
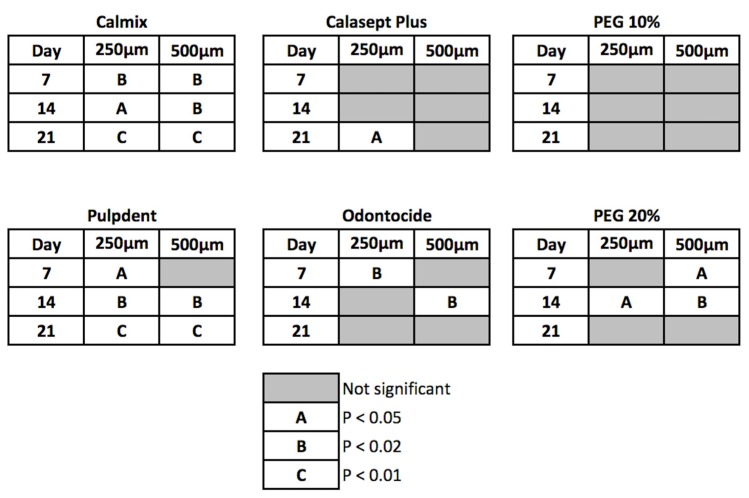
Visual representation of changes from the baseline in pixel data for the red and green channels. Sample areas were located at 250 and 500 μm from the canal wall in the radicular dentine. Data are the mean of three independent experiments. Statistically significant changes, when assessed using ANOVA followed by post-hoc Dunn’s tests, are represented according to the legend.

The differences between treatments were statistically significant at 7 days and beyond for the green and red channel data, with Calmix™ superior to the other groups. After 7 days, the colour change continued to rise slowly for Calmix™ but began to decline for Odontocide™, indicative of the buffering of hydroxyl ions by dentine. A summary of the statistical significance of the change over time is given in Figure 4.

Of the groups showing significant changes in both green and red pixel data at 250 μm from the canal wall, Calmix™ and Pulpdent™ produced a change at day 7 which was sustained throughout the remainder of the study period. In contrast, Odontocide™ demonstrated a significant change from the baseline at day 7, but this was not maintained over time. Likewise, the paste comprising 20% calcium hydroxide in PEG 400 caused a change at day 14 but this was not sustained thereafter. Calasept Plus™ did not give a significant change until day 21.

At the 500 μm depth from the canal wall, both Calmix™ and 20% calcium hydroxide in PEG 400 were the first groups to show a significant change at day 7. This was followed by Pulpdent™ at day 14. The changes in both Calmix™ and Pulpdent™ were sustained throughout the rest of the study. Odontocide™ showed a significant change at the 14-day mark but this was not sustained thereafter. The paste containing 20% calcium hydroxide in PEG 400 no longer had a significant change after day 21. With Calasept Plus™, there was no significant change from baseline throughout the entire experiment.

## 4. Discussion

The results of this study show variations between the hydroxyl ion release dynamics of different commercial calcium hydroxide pastes. The commercial pastes used in this study vary in composition, in terms of the solvents and thickeners, and in the loading of calcium hydroxide, with two based on water, one on PEG, water and ibuprofen, and one on PEG alone.

Anthocyanin dye, extracted from red cabbage, has been used as a pH indicator in the food science industry, including within food labels [11]. Anthocyanin dye was chosen as the indicator for this study because colour changes correspond to the pH changes in a predictable manner. It has a distinct but gradual change between colours, from purple-blue to green and as the pH increases into the highly alkaline range, to yellow, as seen in the prepared buffered pH solutions. The colour changes in the dye are due to the presence of phenolic or conjugated substances, such as cyanidin, delphinidin, pelargonidin, peonidin and petunidin, which undergo structural changes according to variations in pH [12].

Because the colour changes in pH dyes are reversible, fluctuations in the pH due to dentine buffering can be seen. The buffering effects of dentine proteins have been demonstrated in previous studies, where dentine powder was added to saturated aqueous solutions of calcium hydroxide [13]. In the present study, Calmix, which uses a water-free vehicle, produced a sustained effect for 3 weeks that extended up to 500 μm from the canal wall, and those effects did not show a reversal over time. In contrast, with Odontocide™, colour changes were reversed after one week at 250 and 500 μm from the canal wall, indicating that buffering by dentine had occurred. This difference could be due to several factors. Firstly, the vehicle of Odontocide is PEG mixed with water and the water is 5% w/w of the product. Secondly, Odontocide also contains ibuprofen (isobutylphenylpropionic acid), which as a carboxylic acid (pKa of 4.4) could influence the overall pH of the medicament, since it behaves as a weak acid and its solubility is affected by pH as well as by viscosity [14]. The relapse of the pH is noted when compared with the 20% calcium hydroxide + PEG 400 group, which has the same concentration of calcium hydroxide but PEG 400 only as a solvent.

The presence of water in the solvent for calcium hydroxide has been thought to cause more rapid release than more viscous vehicles [2]. Adding to this, the present results show variations between materials where the solvent is water, namely Pulpdent™ and Calasept™, where the major difference in composition is the presence of carboxymethyl cellulose in Pulpdent™ as a thickening agent. The manufacturer’s information for Calasept Plus™ does not disclose the presence of any viscosity modifiers in this material.

The pH changes are a result of the interplay of hydroxyl ion diffusion with the buffering actions of dentine. The gradient of ions is greatest where the hydroxyl ions have been buffered by the protons, which are released from the carboxyl groups of amino acids from dentine proteins. The gradient is influenced by the viscosity of the medicament paste (which affects the rate of diffusion), the concentration of available (soluble) hydroxyl ions and the extent of any reservoir of undissolved calcium hydroxide. In terms of the dentine tubules, moving from the pulp towards the outer root surface, the dentine tubules change in diameter and direction, with resultant changes in the surface area for interaction with released ions. There is less intertubular dentine and wider tubules near the pulp [15]. Thus, there is a unique pattern of interactions across the thickness of the root for each calcium hydroxide medicament. This explains the differences between the various medicaments examined at any given point in time and at one specified distance from the root canal wall.

For effective antimicrobial activity, hydroxyl ions must be released from calcium hydroxide [16,17]. To achieve worthwhile actions, there must be a sufficient quantity of hydroxyl ions to overwhelm the buffering effects of the dentine, so that a sufficiently alkaline pH environment can be created and sustained within the root canal system and within the adjacent dentine. Achieving high concentrations of hydroxyl ions is essential, as *Enterococcus faecalis* can tolerate an environment with a pH of 10 but is inactivated at pH values around 11.5 to 11.9 [18,19].

The solubility of calcium hydroxide is greater in PEG 400 than in water and therefore, more hydroxyl ions are available to diffuse into the inner radicular dentine. The molecular structure of PEG consists of a large number of ethylene oxide groups along its backbone, allowing it to form complexes with metal cations, such as calcium ions [20]. The binding of calcium ions drives the dissociation of calcium hydroxide, thus releasing more free hydroxyl ions. As predicted, this study demonstrates that the PEG 400-based material Calmix™ was successful at prolonging the effect at depths up to 500 μm over time.

In terms of clinical significance, achieving a large and sustained alkaline pH effect is desirable, as this enhances antimicrobial activity. Commercial calcium hydroxide pastes differ in the extent of pH change they induce in the radicular dentine. When the pH changes reverse from dentine buffering, the therapeutic effects decline, so placing the paste for an extended period of time may not be beneficial. A final practical point is that existing pastes vary in their viscosity and thus, how well they can be delivered through fine nozzles. The flow characteristics and viscosity of PEG-based materials makes them suitable for delivery through a fine tip, and for direct delivery towards the root apex from a syringe with a small tip. When PEG is used alone and no water is present, the material will not dry out over time within the delivery system.

## 5. Conclusions

This laboratory study has shown variations between calcium hydroxide materials, where hydroxyl ions alter the pH within the radicular dentine. These effects reflect differences in composition, namely the choice of solvent and the inclusion of viscosity modifiers. Using PEG 400 as a vehicle for calcium hydroxide has demonstrated sustained diffusion of hydroxyl ions when compared to PEG 400 with water. Likewise, altering the viscosity of water-based calcium hydroxide pastes altered the effects seen from released hydroxyl ions. Overall, pastes which used a completely waterless solvent demonstrated greater alkaline pH changes in radicular dentine than those containing water.

## Figures and Tables

**Table 1:** Components of commercial calcium hydroxide pastes.

Parameter	Pulpdent™	Calasept Plus™	Calmix™	Odontocide™
Active ingredients	39–42%	41.07%	37.5%	
Calcium hydroxide				20%
Other				7% Ibuprofen
Vehicle	Water	Isotonic saline	PEG 400	PEG 400 with water
Thickener	Methyl cellulose	None	PEG 4000	PEG 3000
Radiopaque agent	Barium sulphate	Barium sulphate	Zirconium dioxide	Barium sulphate
Batch number	120213	0350	08042015	6002

Calcium hydroxide composition is shown in percent by weight. The information on product composition was obtained from material safety data sheets and manufacturer websites. There is no thickener disclosed for Calasept Plus™ by the manufacturer. PEG = polyethylene glycol.
